# Parent-Infant Interactions Among Infants With High Risk of Cerebral Palsy: A Protocol for an Observational Study of Infant and Parental Factors for Dyadic Reciprocity

**DOI:** 10.3389/fpsyt.2021.736676

**Published:** 2021-09-29

**Authors:** Katrine Røhder, Maria Willerslev-Olsen, Jens Bo Nielsen, Gorm Greisen, Susanne Harder

**Affiliations:** ^1^Department of Psychology, University of Copenhagen, Copenhagen, Denmark; ^2^Elsass Foundation, Charlottenlund, Denmark; ^3^Department of Neuroscience, Panum Institute, University of Copenhagen, Copenhagen, Denmark; ^4^Neonatal Department, Rigshospitalet, Copenhagen, Denmark

**Keywords:** cerebral palsy, parent-infant interaction, dyadic reciprocity, parental representations, WMCI, parental mental health, infant interactive behavior

## Abstract

**Background:** An early diagnosis of chronic disability, such as risk of Cerebral Palsy (CP), is likely to affect the quality of parent-infant interactions by affecting both infant and parental factors. Due to adverse perinatal events, infants at high risk of CP may exhibit less engagement in interactions, while parents may experience increased mental health problems and disrupted parental representations that can have a negative effect on parental sensitivity. Recent clinical guidelines on early intervention among families with infants at risk of CP recommends supporting parental sensitivity and mutual enjoyable interactions more research is needed to inform such interventions. This includes understanding how infant and parental risk as well as resilience factors impact parent-infant interactions and how existing parenting programs developed among typical developing infants should be adapted to families with infants at risk of CP. In addition, as majority of research on infant neurohabilitation focus on improving motor and cognitive outcomes research on infant emotional development is needed. The study aim is to assess the quality of early parent-infant interactions in families with high-risk infants, compared to families with low-risk infants, and to explore how interaction quality is affected by infant and parental factors. Three potential mediating factors explaining the association between CP risk and less optimal parent-infant interactions will be explored: infant interactional capacities, parental mental health and well-being, and parents' representations of their child.

**Methods:** The prospective, longitudinal design will follow infants at high risk for CP and their parents and a control group at three time points from 15 weeks to 15 months corrected infant age (CA). Measures comprise infant developmental assessments, questionnaires and interviews with both parents, and global ratings of video-recorded parent-infant interactions.

**Discussion:** Study results will enhance our understanding of how parent-infant interactions may be affected by perinatal neurological risk and identify potential important mechanisms for observed associations. This knowledge could assist in planning future early screening and intervention programs and identifying families who should be offered targeted psychological interventions in addition to neurohabilitation programs.

## Introduction

Cerebral palsy (CP) is the most common physical disability during childhood, affecting two in 1,000 children (1, 2). CP is a neurodevelopmental disorder primarily affecting movement and posture and limiting activity due to non-progressive disturbances in the developing fetal or infant brain (3). Children with CP experience a range of difficulties affecting their level of function. Forty percent of children with CP are unable to walk independently (4, 5), language impairment is common, with one-third of affected children being non-verbal (6, 7), 28% have musculoskeletal deformities (e.g., hip displacement), 11% have vision impairments, and 4% hearing impairments (8). Half (49%) of all children with CP experience intellectual disability and 26% experience emotional and behavioral disorders during childhood (9–11).

Its etiology is complex and not fully understood, but CP is associated with prenatal factors such as pre-term birth (in 30–50% of affected children), fetal growth restriction, pregnancy disorders (pre-eclampsia, placenta abruption, chorioamnionitis), and major birth defects (12–14). Birth asphyxia is estimated to account for <10% of cases (15).

Severe perinatal brain injury is usually diagnosed at or shortly after birth, carries a high risk of CP, and constitute a significant fraction of the etiology of CP. However, CP may not be detected until much later in children with less severe impairments. In a Danish study, average age at diagnosis was 1 year (16). Late diagnosis precludes intervening when the nervous system is most plastic and interventions may have the greatest impact on infant development (8, 17–19). Recent advances in diagnostics enable identifying infants at high risk for CP before the age of 5 months. Diagnostic indicators include a combination of neonatal magnetic resonance imaging (MRI), standardized motor assessment, such as the Prechtl Qualitative Assessment of General Movements (GMA), and clinical assessment of risk factors (18, 19). Early detection makes it possible to intervene at a very early stage with intensive neurohabilitation consisting of daily home-based training in stimulating environments, which has a more beneficial impact on infant cognitive and motor development than standard physiotherapy alone (17, 19, 20). Thus, it is essential that children at risk for CP are offered intensive neurohabilitation training as early as possible.

Actively involving infant and parents in neurohabilitation activities is crucial for infant developmental outcomes (i.e., outcomes relying on infant-initiated activities and daily parental coaching). To some extent, the quality of neurohabilitation depends on infants' abilities to engage in interactions and parents' abilities to read and respond appropriately to infant initiation (i.e., parental sensitivity). This study seeks to examine the early interactions of parents and their infants who are at risk for CP. While parents can benefit from advice and emotional support, there is also a risk of stressing their relation to the child with an uncertain future, to establish a stigma, and increasing guilt by setting goals that are not achievable. The resulting knowledge could assist in planning future early screening and intervention programs by providing insight into optimal parent involvement strategies in early neurohabilitation and helping to identify families at risk for suboptimal parent-infant interactions and offering targeted psychological interventions in addition to neurohabilitation.

An additional study aim is to examine the understudied domain of socio-emotional development of infants at risk for CP. Research among typical developing infants has shown that healthy infant socio-emotional development is associated with better social functioning in later childhood and decreased risk of emotional and behavioral problems (21). Socio-emotional development may be directly affected by adverse perinatal events and indirectly by parental distress and difficulty adjusting to parenthood. It is important to describe the prevalence of healthy socio-emotional functioning among infants at risk of CP and to elucidate potential associations between adverse perinatal events, parent-infant interactions, and infant socio-emotional development, potentially establishing the importance of supporting socio-emotional development of the child and parental well-being.

### Parent-Infant Interactions

A recent systematic review found that families with infants at risk for CP have fewer optimal and more disrupted parent-child interactions during the first year of life, compared to families of low-risk infants (22). Compared to low-risk infants, high-risk infants are more likely to be described as less engaged, less active and more fretful. Their parents are at greater risk of being less emotionally involved with and less sensitive to the infants and more intrusive, inappropriately stimulating their infants more than mothers of developmentally typical or low-risk infants. The authors suggest that the severity of the infants' medical conditions leads to suboptimal infant interactive behavior and increased maternal mental health problems (depression, distress, and anxiety). These factors may negatively influence mothers' interactive behavior. However, many of the studies included in the review used pre-maturity and low birth weight as primary selection criteria, yielding heterogeneous sample populations and limiting conclusions about infants at high risk for CP based on the recent recommendations regarding early diagnosis. Festante et al. (22) recommend conducting longitudinal studies among more homogenous samples (e.g., infants with absent fidgety movements associated with high risk of CP vs. heterogeneous samples of pre-mature infants).

Other studies have examined and found qualitative and diagnostic specific differences in parent-infant interactions in specific populations, such as infants at risk of autism or infants with Downs Syndrome, compared to typical developing infants (23, 24). Adamson et al. (25) compared infants at risk of autism, infants with Downs Syndrome, and typical developing infants and found that infants at risk of autism displayed less engagement in social interactions, while infants with Downs's syndrome displayed a heightened and prolonged social attention - both compared to typical developing infants. Similarly, Field (26) found that children with disabilities, including those with CP, have more difficult temperaments than children with delayed development who in turn had more difficult temperament than developmentally typical infants and infants with Down's syndrome. These studies support the need for observational studies examining how specific characteristics of different disorders affect parent-infant interactions. The primary characteristic of CP is atypical motor development that may have a specific impact on the quality of parent-infant interactions by limiting movement and interactive touch but other comorbid features or related aspects of CP may also have an impact on interactive abilities. Furthermore, parents of infants at risk of CP must deal with early, intense experiences of pregnancy and childbirth as well as uncertainty about the child's future development, both of which are likely to affect parental mental health and parental behavior.

**Figure 1:**
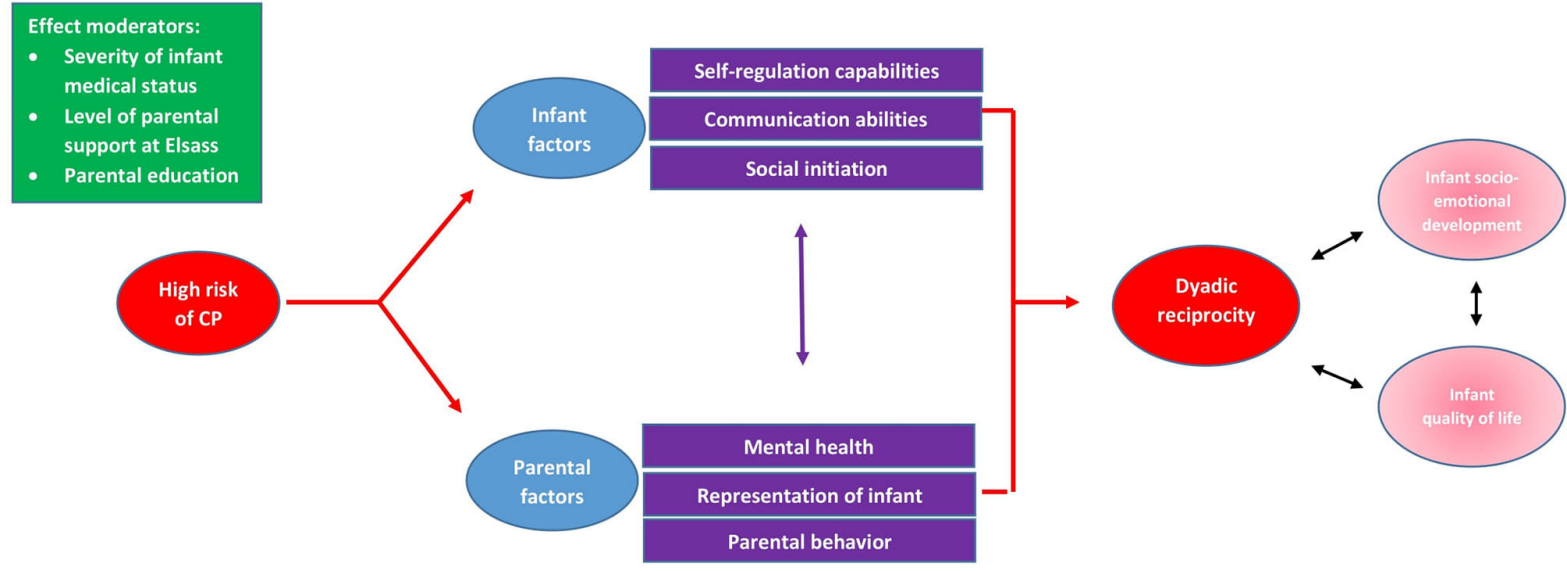
Study Model.

In order to develop targeted early intervention programs to support parents of infants at high risk of CP, we want to examine risk and protective factors for non-optimal parent-infant interactions. In accordance with the literature from both risk and non-risk populations, parental mental health difficulties [e.g., depression, anxiety, stress, and birth-related trauma; (27, 28)], infant interactive behavior [e.g., initiation, communication and self-regulation; (29–31)], and parental representations of the infant (32) will be examined as main risk factors for non-optimal dyadic interaction (see Figure 1 for study model).

### Fathers

Research on infants at risk for CP has been performed almost exclusively among mothers and infants. To the best of our knowledge, only a single study included fathers. Feldman (33) found both that paternal involvement in child caregiving reduced maternal distress and no differences between father-infant and mother-infant interactions among families with pre-mature and low birth weight infants. Consistent with current recommendations (34), we consider both parents to be part of infants' daily lives and will include observations of both fathers/co-parents and mothers with infants.

## Methods and Analysis

### Aims and Hypotheses

The primary study aim is to assess whether parental interactions with high-risk infants are less optimal than parental interactions with low-risk infants and to describe potential difficulties in interactions between parents and infants at high risk for CP. Secondly, we aim to explore infant and parental risk factors that may mediate the expected effect of neurological risk on interactional quality. We are particularly interested in exploring three inter-related mechanisms: (1) infant interactional behavior, (2) parental well-being, and (3) parental representations of the child. Third, we want to investigate whether dyadic reciprocity at 15 months is directly associated with infant socio-emotional development and infant quality of life. Fourth, we want to examine developmental trajectories of socio-emotional development in infants at high-risk of CP from 15 weeks corrected age until 4 years of life. Finally, as families with infants at high-risk of CP are enrolled in a randomized controlled trial we exploratively want to examine whether families receiving intervention or usual care differ on psychological measures.

We hypothesize that:

1. Interactions between parents and infants at high risk for CP at 15 months corrected age (CA) are characterized by inferior dyadic reciprocity, compared to interactions between parents and low-risk infants.
2. Infant and parental factors mediate the expected association between high risk of CP and the hypothesized inferior dyadic reciprocity at 15 months. Specifically, we expect high-risk infants to have inferior self-regulation abilities and restricted communication abilities and to be less involved in interactions with parents than low-risk infants and that these factors are associated with less dyadic reciprocity at 15 months. Furthermore, we expect parents of high-risk infants to experience lower levels of parental well-being (i.e., increased mental health symptoms and parental stress), to have more unbalanced representations of their child, and to exhibit less sensitivity and more intrusiveness in interactions with their child, compared to parents of low-risk infants. Further, we expect these factors to negatively affect the quality of dyadic reciprocity. *Post-hoc* analyses will explore the relative contribution of measured factors to dyadic quality, the importance of specific age periods (sensitive periods) to dyadic reciprocity at 15 months and differences between mothers and fathers/co-parents.
3. Dyadic reciprocity, infant socio-emotional development, and infant quality of life are associated.
4. Infants at risk of CP show atypical or delayed socio-emotional development compared to low risk controls.

We do not have any hypothesis regarding differences between groups enrolled in the RCT as participating in an intensive early intervention program can have both a positive and negative affect on parents and their interactional capacities.

### Design

The prospective, longitudinal cohort design includes a comparison group. Infants at high risk for CP and their parents are followed from 15 weeks to 4 years corrected age (CA). The comparison group consists of infants at low neurological risk and their parents. Six assessment points are planned: 15 weeks (T1), 9 months (T2), 15 months (T3), 2 years (T4), 3 years (T5), and 4 years (T6) CA.

The study is part of a research collaboration between the University of Copenhagen Departments of Psychology and Neuroscience and the Elsass Foundation, a private non-profit organization offering support to people living with CP and their families. It is being conducted in conjunction with a randomized controlled trial (RCT) examining the effectiveness of an early intervention program to support infant muscle growth and mobility and to prevent contractures in which high-risk infants and their families are enrolled (35).

### Participants

Infants at high risk for CP are recruited from all neonatal wards from the Capital Region and Region Zealand. Following recent recommendations (19), we define high risk of CP with the following inclusion criteria: (1) infant CA <17 weeks; (2) suspected brain lesion on the basis of medical assessment, MRI or ultrasound findings, or absence of fidgety movements, as determined by the GMA; and (3) brain lesion severity determined by clinicians to warrant informing parents about the associated risk for CP. Low-risk infants will be recruited by health nurses at home visits from two Danish municipalities (one rural, one urban). In Denmark, families with low-risk births are discharged home 4 h after birth, precluding recruitment at the hospital. Infants and their families will be included in the control group if there is no evidence of an infant brain lesion within 17 weeks after birth or known perinatal adverse events, such as pre-term birth (<36 weeks GA). Exclusion criteria for all infants are severe genetic abnormalities, severe heart problems, metabolic diseases, or ongoing hospitalization.

### Procedures

All parents of eligible high-risk infants are informed about the study only after they have discussed their child's risk status with their medical team. Recruitment takes place from Fall 2020 through Winter 2023.

Families receive written information regarding the study's purpose and design and have an opportunity to speak with the investigators before consenting to the study. After consent is obtained, the family is invited to visit the Elsass Foundation, where data collection takes place at planned infant ages.

In the RCT, infants at high risk for CP are randomized to a 6-month intensive home-based intervention between T1 and T2 or usual care. Due to distressing perinatal experiences among families with infants in the high-risk group, all parents of high-risk infants are offered a brief psychological intervention of up to five supportive conversations with a psychologist at Elsass. Conversations focus on parents' thoughts and feelings related to the birth and early post-partum period, including receiving the information that their infant is at neurological risk. These conversations take place only after T1.

Data collection will consist of a combination of developmental assessments of the infant, questionnaires to and interviews with both parents, and video-recordings of parent-infant interactions (see Table 1).

**Table 1:** Measures used in the study.

	**15 weeks**	**9 months**	**15 months**	**2 years**	**3 years**	**4 years**
Infant	Bayley-III	Bayley-III	Bayley-III			
	AIMS	AIMS	AIMS			
	ITQOL	ITQOL	ITQOL	ITQOL	ITQOL	ITQOL
	ASQ-SE2	ASQ-SE2	ASQ-SE2	ASQ-SE2	ASQ-SE2	ASQ-SE2
Both parents	Socio-demographic information					
	WMCI		WMCI			
	PSI-SF	PSI-SF	PSI-SF	PSI-SF	PSI-SF	PSI-SF
	DASS-21	DASS-21	DASS-21	DASS-21	DASS-21	DASS-21
	EPDS	EPDS	EPDS			
	City BiTS	City BiTS	City BiTS			
	Parent-infant face-to-face interaction (CIB)	Parent-infant face-to-face interaction (CIB)	Parent-infant face-to-face interaction (CIB)			
Mother only	Feeding interaction (CIB)	Feeding interaction (CIB)	Feeding interaction (CIB)			
Father or coparent only	Diaper-changing interaction (CIB)	Diaper-changing interaction (CIB)	Diaper-changing interaction (CIB)			

*AIMS, Alberta Infant Motor Scale; ASQ-SE2, Ages and Stages Questionnaire–Social Emotional; CIB, Coding Interactive Behavior; City BiTS, City Birth Trauma Scale; DASS-21, Depression, Anxiety, and Stress Scales-21; EPDS, Edinburgh Postnatal Depression Scale; ITQOL, Infant/Toddler Quality of Life Questionnaire; PSI-SF, Parenting Stress Index-Short Form; WMCI, Working Model of the Child Interview*.

### Primary Outcome

#### Dyadic Reciprocity

The primary outcome of dyadic reciprocity is measured by coded video recordings of parent-infant interactions. The Coding Interactive Behavior Manual (CIB) provides six composite scores for parental sensitivity and intrusiveness, infant involvement and withdrawal, and dyadic reciprocity and negative states, calculated as means of item scores (36). Dyadic reciprocity consists of three items (reciprocity, adaptation-regulation, and fluency) and is coded at the dyadic level. High scores represent good dyadic reciprocity characterized by harmonious give-and-take interactions in which both infant and parent are engaged and contribute to the mutual exchange (37). A separate scale applied for feeding interactions provides four additional scales: distractibility, independence, negotiation during feeding, and feeding efficacy. The CIB has been validated in several studies across cultures, infant age groups, and parental gender and can differentiate between different high-risk groups (e.g., parental mental health issues and pre-mature birth) and control groups (33). Interactions are coded blinded to group status, with inter-coder agreement calculated on a randomly selected 20% of interactions.

Varying situations affect interactional quality, and including different contexts when observing parent-infant interactions is recommended (38, 39).

Five minutes of mother-infant and father-infant face-to-face interaction are video recorded at T1 and T2 when infants are calm and alert, far from feeding time, and without pacifier use. Infants are placed in an infant chair with each parent in turn seated opposite and leaning toward the infant at a distance of 30–40 cm. No toys are used during the interaction. Two cameras synchronized and placed laterally to the dyad film the infant's face and upper body and the parent's face and upper body. At T3, 5 min of mother-infant and father-infant free-play interaction are video recorded. Parents are asked to freely interact or play with the child on a carpet supplied with age-appropriate toys. The use of toys during the interaction is not mandatory and parents and infant can move around the carpet throughout the interaction. A camera placed frontally or laterally to the dyad records interactions. The whole scene is video recorded and, unlike face-to face interaction, with no particular focus on details about participants' faces and bodies.

In addition to face-to-face and free-play interactions, mother-infant feeding interactions and father-infant diaper-changing interactions are recorded at T1, T2, and T3. Mothers are asked to feed their infant as they normally would (i.e., breast or bottle feeding or solid food, depending on preferences and infant age). Fathers are asked to interact with their child when changing a diaper as they normally would. Interactions are video-recorded and their duration is noted.

### Secondary Outcomes

Our hypotheses about mediating factors are assessed with both infant and parents. Infant factors include parent-reported self-regulation abilities, observational measures of interaction engagement with parents, and standardized assessment of communication abilities. Parental factors include representations of the infant, self-reported mental health and well-being, and observations of parental interactional sensitivity and intrusiveness.

#### Infant Self-Regulation Abilities

The Ages and Stages Questionnaire–Social Emotional (ASQ-SE2) is used to assess self-regulation (40). The ASQ-SE2 is a short questionaire assessing parental reports of infant self-regulation, compliance, communication, adaptive functioning, autonomy, emotions, and interaction with other people. Age group-specific versions are available, and the version for infants aged 3–8 months is used at T1 and T2 and the version for infants aged 9–14 months at T3. Parents are asked to indicate frequency of and any concerns about 18 items describing age-appropriate infant behaviors. In addition, parents are asked to freely elaborate on potential concerns. The ASQ-SE2 has acceptable reliability and is one of the most comprehensive and psychometrically sound measures of infant socio-emotional development (41).

#### Infant Involvement

The involvement of the infant in interactions with each parent at T1 and T2 is assessed with the CIB composite of infant involvement (36). It consists of six items: infant gaze/joint attention, infant positive affect, alert, fatigue (revised), vocalization, and infant initiation. High scores represent high involvement in interactions.

#### Infant Communication Behavior

The language/communication subscales of the third edition of the Bayley Scales of Infant and Toddler Development (Bayley-III) are used at T1, T2, and T3 (42). The Bayley-III is a standardized individual assessment of infant performance that is completed with the parent present. Language evaluation consist of two subscales: receptive and expressive communication. The Bayley is specific to developmental stages, and the infant is presented with age-related toys and tasks to perform. American norms are available. The Bayley-III has been assessed among low-risk Danish children, demonstrating both cultural differences and predictive validity (43, 44).

#### Parental Representations of Their Child

The Working Model of the Child Interview (WMCI) is used at T1 and T3 (45). The WMCI is a semi-structured, open-ended parental interview; completion takes ~1 h. Interviews are video recorded and subsequently coded. Four types of representations are summarized to reflect parents' current state of mind about their infant: balanced, disengaged, distorted, and, through the additional WMCI-D scale, disrupted (46). Balanced representations (i.e., the parent is engrossed with the child and conveys an appreciation of the child's subjective experiences) is related to secure infant attachment. In contrast, unbalanced representations are disengaged, distorted, or disrupted. Disengaged representations (i.e., the parent is emotionally distant from and sometimes describes aversion to the child) are associated with insecure-avoidant infant attachment, while distorted representations (i.e., the parent is pre-occupied with other concerns, such as mental health problems, and gives incoherent, unrealistic, confused descriptions of the child) are associated with insecure-ambivalent attachment (32). Disrupted representations are characterized by parental fear or frightening behavior, role-boundary confusion, affective communicative errors, hostility, and parental withdrawal from the child and are associated with insecure-disorganized attachment. The WMCI has been found to be a reliable and valid approach to scoring representational aspects of parent-child relationships (47–52).

#### Parental Sensitivity and Intrusiveness

The parental codes of the CIB are used to assess recorded interactions with the infant. Parental sensitivity consists of 10 items: acknowledging, imitating, elaborating, parent gaze/joint attention, positive affect, vocal appropriateness, appropriate range of affect, resourcefulness, affectionate touch, and parent supportive presence. Parental intrusiveness consists of five items: forcing, overriding, parent negative affect/anger, hostility, parent anxiety.

#### Parental Mental Health and Well-Being

At T1-T6, parental mental health is assessed with the Depression, Anxiety and Stress Scales-21 (DASS-21), a 21-item questionnaire assessing symptoms of depression, anxiety, and stress during the past week (53, 54). Possible item scores range from 0 (did not apply to me at all) to 4 (applied to me very much, or most of the time). The DASS-21 is widely used and has been applied in earlier studies among parents of infants with CP (55). It has good psychometric properties (56–58).

#### Parental Birth-related Post-traumatic Stress Disorder

Birth-related trauma among parents will be assessed with the City Birth Trauma Scale (59), a 29-item questionnaire developed to measure birth-related trauma using DSM-5 diagnostic criteria for PTSD. Items are rated on a 4-point scale for frequency during the previous week (0, not at all; 1, once; 2, 2–4 times; and 3, 5 or more times). Four clusters of PTSD symptoms are assessed (re-experiencing, avoidance, negative cognitions and mood, and hyperarousal) as are symptoms of dissociation during birth. Psychometric studies (60, 61) have confirmed the presence of two factors: birth-related PTSD symptoms of intrusion, avoidance, and negative cognitions and mood specifically related to birth, and general PTSD symptoms of negative cognitions and mood and hyperarousal. The City Birth Trauma Scale has good psychometric properties and has been validated in several languages. The current study uses a Danish translation of the scale.

#### Parental Stress

To assess parental stress, the fourth edition of the short-form Parenting Stress Index-Short Form (PSI-4-SF) is used at T1-T6 (62). The PSI-4-SF comprises three subscales—parental distress, parent-child dysfunctional interaction, and difficult child—and a total stress scale. Studies provide psychometric support for the PSI-4-SF as an effective and appropriate measure for use with high-risk families (63). The PSI-4-SF has been used among parents with children with CP (64).

#### Postnatal Depression

At T1-T3, the Edinburgh Postnatal Depression Scale (EPDS) is used with parents (65). The EPDS consists of 10 items assessing depressive symptoms during the past 2 weeks and is a valid screening instrument for postnatal depression. A cut-off score of 11 for detecting clinical depression was recently validated among Danish mothers of infants, with sensitivity and specificity of 77 and 90% for DSM-V diagnostic criteria and 80 and 96%, for ICD-10 criteria (66).

### Tertiary Outcomes

#### Infant Socio-Emotional Development

The ASQ-SE2, described above under infant self-regulation abilities, is used at T1-T6 to assess overall socio-emotional development.

#### Infant Quality of Life

At T1-T6, the quality of life of participating high-risk infants is assessed with the Infant/Toddler Quality of Life Questionnaire (ITQOL) (67). The ITQOL was developed using the World Health Organization definition of health as a state of complete physical, mental, and social well-being and not merely absence of disease (68). It consists of 47 items assessing physical function, growth and development, bodily pain, temperament and moods, behavior, and general health perceptions, as well as how infant health or handicap affects parental and family well-being. Response options use a five-point Likert scale, with higher scores indicating better functioning. Mean scores are calculated to derive an overall scale score. The ITQOL has good reliability and validity (69).

### Effect Moderators

#### Severity of Infant Medical Condition

Infants' medical condition at birth is assessed through electronic birth health records. In addition, infant cognitive and motor development trajectories are assessed with the cognitive scale of the Bayley-III, described above under infant communication behavior, and the Alberta Infant Motor Scale (AIMS) (42, 70), a standardized scale assessing delayed and abnormal motor development in infants. AIMS can be used from birth to 18 months and takes 20–30 min to complete. Infant movement is assessed in prone, supine, sitting, and standing positions. AIMS is easy to administer and focuses on evaluating motor milestones and the quality of posture and movements and has superior predictive validity than other assessments of motor development (70).

#### Parental Education and Family Relationships

The educational level of parents and their parity is explored as effect moderators. At T1, parents self-report information regarding education, employment, family relationships (including other children), and annual family income.

#### Level of Parental Support

The degree of support received by parents is measured as randomization to the intervention group, measured dichotomously as yes/no, and the degree to which parents received the supportive intervention with a psychologist at Elsass during the observation period, measured as number of hours.

### Statistical Analysis

The primary outcome is between-group differences in dyadic reciprocity at 15 months CA. The statistical significance of any differences is assessed with *t*-tests for parametric data and the Mann-Whitney test for non-parametric data. Regression analysis and mediation analytical approaches explore the impact of infant and parental factors and interactional mechanisms.

Based on prior research (33), medium to high effect sizes are expected. Medium effect sizes can be detected at a 5% level of significance and a power of 80% with a sample size of 198, with 99 families in each group to detect between-group differences. High effect sizes can be detected with a sample size of 38, with 19 families in each group. For regression analyses, expecting small effect sizes, 5% significance level, 80% power, and five predictors (one infant, two parental, and two confounders), a total sample size of 92 is required.

Infants at high risk of CP are relatively uncommon and may be difficult to recruit. Consequently, we realistically aim to recruit 92 families, with 46 in each group. Experiences from a prior Elsass Foundation research project recruiting from a single hospital in the Capital Region suggest it is possible to recruit ~1 family with a high-risk infant per month. We include all neonatal wards in two regions to increase recruitment rates. Recruiting an average of 2.5 families per month will yield a sample size of 92 families within 3 years. Due to the absence of pre-defined hypotheses and the small expected number of families in the two randomization groups, any comparison between them will be exploratory, only.

## Discussion

Study findings will enhance our understanding of how parent-infant interactions may be affected by the infant's neurological risk status and identify important mediating factors related to infant interactive behavior, parental well-being, and parental representations of the child. This knowledge can assist in planning future interdisciplinary, relationship-focused neurohabilitation programs for infants at high risk of CP and their families that include supporting the emotional development of high-risk infants and the well-being of their parents.

### Infant Interactive Behavior

Bidirectional and reciprocal models of parent-child interactions have long been acknowledged as the standard for parent-infant research (71, 72). Such models supersede previous unidirectional models describing how parental behavior affects child development. In bidirectional models, both child and parent can assume the roles of initiator and responder and the relationship is mutually responsive and reciprocal. Adverse perinatal experiences and the risk of a severe neurodevelopmental disorder, such as CP, are likely to restrict infant interactive behavior due to delayed or atypical development in social, communication, and self-regulation abilities.

Social initiations, joint attention, and responsiveness to social cues are less frequent among children with disabilities than among typical developed children (73). During the first 6 months after birth, infants at risk of CP are more likely to be less active and engaged in interactions, more fretful, less alert and focused, and less responsive (e.g., displaying fewer facial expressions and more negative engagement cues) than typical developing controls. At 6–12 months, during play interactions, high-risk infants show less exploratory behavior and are more dependent on their mothers' structuring strategies being more passive and avoidant at play compared to developmentally typical children. More severe neurodevelopmental illness is associated with less interactive behavior (22). In addition, several studies have found that infant restricted communication abilities are associated with poorer interaction quality with caregivers. Pennington and McConachie (29) studied children aged 2–10 years with CP and found that child expressive language abilities were the main predictor of restricted communication patterns with parents, characterized by less child initiation and responses. Similar results were found by Harel-Gadassi et al. (31) studying pre- and term born infants. Finally, infants born pre-maturely are more likely to have self-regulation difficulties than are infants born at term (74). As such, Feldman (33) found that infants born pre-maturely with very low birth weight [<1,650 g, gestational age (GA) < 33 weeks] and those with intrauterine growth retardation, who are both are at risk of CP (15), showed more negative emotionality in interactions with both mother and father, compared to interactions in families with infants at low risk and families with mothers with postnatal mental health problems. Likewise, Liu et al. (75) found that during the first 2 months of their lives, infants diagnosed with CP at the age of 3 years had poorer attention, needed more adult handling to remain calm and alert, lower self-regulation, more arousal excitability, more hypertonicity, and lower quality of movement and exhibited more stress signs than other infants.

Our study will contribute with detailed knowledge on how infant interactive behavior may be affected by the adverse perinatal events associated with CP steaming from both parental-reported and observational accounts. Such knowledge can assist in developing psychoeducative programs and adapting existing early psychological intervention programs to infants at high risk of CP.

### Parental Well-Being

It is well-established that parents of children with CP experience higher levels of parenting stress, anxiety, and depression than do parents of typically developing children (64, 76–79). Our study is the first to also study the prevalance of birth-related parental trauma and the effects of such symptoms on parental behavior. A recent systematic review found that severity of infant disability was associated with higher levels of parental mental health problems among older children with CP, but the review did not assess the timing or cause of parental mental health problems (77). Studies among pre-mature infants have found that 26% of their parents experience clinically significant mental health problems, compared to 12% of parents of term-age infants (80). This highlights the importance of detecting mental health problems among parents of pre-mature infants and examining the influence of parental mental health on parent-infant interactions. Doing so is particularly important among parents with infants at high risk for CP because they face more severe and specific developmental challenges than do families with pre-mature infants. Furthermore, it is important to understand the causality between mental health problems in parents of infants at high risk for CP and difficulties in parent-infant interactions.

### Parental Representations of the Child

A final possible mediator of the association between neurological risk and interactional quality is parental representations of the child. The concept of internal parental representations deriver from attachment theory and have been suggested as an important predictor of infant attachment (47). Beginning in pregnancy, parents develop internal representations of their child that guide their expectations and behaviors in relation to the child. Empirically, maternal representation of the child is associated with maternal behavior (47–50) and infant attachment behavior (51, 52).

Adverse perinatal events, such as pre-mature birth, and infant developmental difficulties (e.g., failure to thrive, sleep disorders, and maltreatment) are associated with an increased risk of unbalanced parental representations of the child [i.e., associated with insecure infant attachment; (32)]. Studies among parents of pre-mature infants have divergent findings. Korja et al. (81), Tooten et al. (82), and Hall et al. (83) found no differences in the risk of unbalanced representations between parents of infants born pre-term and at term. Other studies found that perinatal risk severity was associated with an increased risk of unbalanced or suboptimal parental representations (84–86). Distorted representations are more common among mothers of pre-mature infants, while disengaged representations are more frequent among their fathers (82).

Our study is the first to study parental representations of both mothers and fathers of infants at risk of CP placing the study within an attachment theoretical perspective. International clinical guidelines (8) recommends supporting attachment formation between their parents and their infants at risk of CP during the first year. As little is known about attachment development in relation to CP, results from our study will provide needed knowledge on an important risk factor for insecure attachment.

### Strengths and Limitations

To our knowledge, this will be the first study to longitudinal examine *both* mother and father-interactions in a sample of infants at high risk of CP during the first 15 months of life. A particular strength of the study is our inclusion criteria that are in line with current recommendations of early diagnosis of CP (19) and the longitudinal design. Second, our assessment of parental interactions include both a naturalistic and experimental setup with several time points which can aid in understanding differences in interactive patterns due to parental gender, age, and context. Third, we thoroughly examine infant and parental risk and protective factors to build specific knowledge on *which* families are at risk of non-optimal interactions and more importantly, *why*. Finally, the study is the first to examine parental representations and birth-related trauma in relation to CP.

There are several limitations to our study. First, families with high risk infants are enrolled in a RCT which may affect the results of this study. The RCT aims to examine the effect of an early intervention consisting of supporting parents in providing daily individualized, goal-directed activities, a nutritional supplement, and neuromuscular stimulation to facilitate infant muscle growth. Although the activities and outcomes of the RCT are not directly related to the aims and outcomes of this study, selection bias cannot be ruled out. We will examine differences in all outcomes among families in the high risk groups but have no hypothesis regarding direction of effects. A second limitation is our inclusion criteria and related limited inclusion period of before 15 weeks corrected age. Only including infants at high risk of CP already identified at a very early age limits the generalization of our conclusions to families with infants with CP identified at later ages. However, as we want to study the formation of and interactive pattern of the at-risk parent-infant relationship in a prospective design this is a necessary inclusion criteria.

## Ethics Statement

Study approval was obtained from the Department of Psychology, University of Copenhagen, Institutional Ethical Review Board (Approval number: IP-IRB/02102020) following the 1975 Helsinki Declaration as revised in 2008. Written informed consent to participate in the study was provided by both parents.

## Author Contributions

KR, SH, and GG: conceptualization and draft of work/substantially revised it. KR, SH, MW-O, and JN: design of the work. KR is PI for this study. MW-O is PI for the RCT-study: investigators. KR, MW-O, and JN: acquisition and analysis. All authors have approved the final version of the manuscript, agreed both to be personally accountable for the author's own contributions, to ensure that questions related to the accuracy or integrity of any part of the work, even ones in which the author was not personally involved, are appropriately investigated, resolved, and the resolution documented in the literature.

## Funding

This study was supported by a personal grant from the Elsass Foundation to KR (Grant Number: 20-3-0990).

## Conflict of Interest

The authors declare that the research was conducted in the absence of any commercial or financial relationships that could be construed as a potential conflict of interest.

## Publisher's Note

All claims expressed in this article are solely those of the authors and do not necessarily represent those of their affiliated organizations, or those of the publisher, the editors and the reviewers. Any product that may be evaluated in this article, or claim that may be made by its manufacturer, is not guaranteed or endorsed by the publisher.

## Abbreviations

CP: Cerebral Palsy
MRI: Magnetic resonance imaging
GMA: The Prechtl Qualitative Assessment of General Movements
GA: gestational age
CA: corrected age
RCT: randomized controlled trial
AIMS: Alberta Infant Motor Scale
ASQ-SE2: Ages and Stages Questionnaire–Social Emotional
CIB: Coding Interactive Behavior
City BTS: City Birth Trauma Scale
DASS-21: Depression, Anxiety, and Stress Scales-21
EPDS: Edinburgh Postnatal Depression Scale
ITQOL: Infant/Toddler Quality of Life Questionnaire
PSI-SF: Parenting Stress Index-Short Form
WMCI: Working Model of the Child Interview
WMCI-D: Working Model of the Child Interview- disrupted.

## References

[1] Himmelman K. Epidemiology of cerebral palsy. In: Olivier Dulac M, Harvey BS, (editors). Handbook of Clinical Neurology (Vol. 111). London: Elsevier (2013). 10.1016/B978-0-444-52891-9.00015-4 23622160

[2] Ravn SH Flachs EM Uldall P. Cerebral palsy in eastern Denmark: declining birth prevalence but increasing numbers of unilateral cerebral palsy in birth year period 1986-1998. Eur J Paediatr Neurol. (2010) 14:214–8. 10.1016/j.ejpn.2009.06.001 19564124

[3] Rosenbaum P Paneth N Leviton A Goldstein M Bax M Damiano D . A report: the definition and classification of cerebral palsy April 2006. Dev Med Child Neurol Suppl. (2007) 109:8–14. 10.1111/j.1469-8749.2007.tb12610.x. 17370477

[4] Christensen D Van Naarden Braun K Doernberg NS Maenner MJ Arneson CL Durkin MS . Prevalence of cerebral palsy, co-occurring autism spectrum disorders, and motor functioning - autism and developmental disabilities monitoring network, USA, 2008. Dev Med Child Neurol. (2014) 56:59–65. 10.1111/dmcn.12268 24117446 PMC4351771

[5] Kirby RS Wingate MS Van Naarden Braun K Doernberg NS Arneson CL Benedict RE . Prevalence and functioning of children with cerebral palsy in four areas of the United States in 2006: a report from the autism and developmental disabilities monitoring network. Res Dev Disabil. (2011) 32:462–9. 10.1016/j.ridd.2010.12.042 21273041

[6] Mei C Reilly S Reddihough D Mensah F Pennington L Morgan A. Language outcomes of children with cerebral palsy aged 5 years and 6 years: a population-based study. Dev Med Child Neurol. (2016) 58:605–11. 10.1111/dmcn.12957 26566585

[7] Zhang JY Oskoui M Shevell M. A population-based study of communication impairment in cerebral palsy. J Child Neurol. (2015) 30:277–84. 10.1177/0883073814538497 25051968

[8] Morgan C Fetters L Adde L Badawi N Bancale A Boyd RN . Early intervention for children aged 0 to 2 years with or at high risk of cerebral palsy: international clinical practice guideline based on systematic reviews. JAMA Pediatr. (2021) 175:846–58. 10.1001/jamapediatrics.2021.0878 33999106 PMC9677545

[9] Novak I Hines M Goldsmith S Barclay R. Clinical prognostic messages from a systematic review on cerebral palsy. Pediatrics. (2012) 130:e1285–312. 10.1542/peds.2012-0924 23045562

[10] Parkes J White-Koning M Dickinson HO Thyen U Arnaud C Beckung E . Psychological problems in children with cerebral palsy: a cross-sectional European study. J Child Psychol Psychiatry Allied Discip. (2008) 49:405–13. 10.1111/j.1469-7610.2007.01845.x 18081767

[11] Rackauskaite G Bilenberg N Uldall P Bech BH Ostergaard J. Prevalence of mental disorders in children and adolescents with cerebral palsy: Danish nationwide follow-up study. Eur J Paediatr Neurol. (2020) 27:98–103. 10.1016/j.ejpn.2020.03.004 32327392

[12] Korzeniewski SJ Slaughter J Lenski M Haak P Paneth N. The complex aetiology of cerebral palsy. Nat Rev Neurol. (2018) 14:528–43. 10.1038/s41582-018-0043-6 30104744

[13] McIntyre S Taitz D Keogh J Goldsmith S Badawi N Blair E. A systematic review of risk factors for cerebral palsy in children born at term in developed countries. Dev Med Child Neurol. (2013) 55:499–508. 10.1111/dmcn.12017 23181910

[14] Tronnes H Wilcox AJ Lie RT Markestad T Moster D. Risk of cerebral palsy in relation to pregnancy disorders and preterm birth: a national cohort study. Dev Med Child Neurol. (2014) 56:779–85. 10.1111/dmcn.12430 24621110 PMC4107088

[15] Nelson KB. Causative factors in cerebral palsy. Clin Obstet Gynecol. (2008) 51:749–62. 10.1097/GRF.0b013e318187087c 18981800

[16] Granild-Jensen JB Rackauskaite G Flachs EM Uldall P. Predictors for early diagnosis of cerebral palsy from national registry data. Dev Med Child Neurol. (2015) 57:931–5. 10.1111/dmcn.12760 25855100

[17] Hadders-Algra M. Early diagnosis and early intervention in cerebral palsy. Front Neurol. (2014) 5:185. 10.3389/fneur.2014.00185 25309506 PMC4173665

[18] Herskind A Greisen G Nielsen JB. Early identification and intervention in cerebral palsy. Dev Med Child Neurol. (2015) 57:29–36. 10.1111/dmcn.12531 25041565

[19] Novak I Morgan C Adde L Blackman J Boyd RN Brunstrom-Hernandez J . Early, accurate diagnosis and early intervention in cerebral palsy: advances in diagnosis and treatment. JAMA Pediatr. (2017) 171:897–907. 10.1001/jamapediatrics.2017.1689 28715518 PMC9641643

[20] Spittle A Orton J Anderson PJ Boyd R Doyle LW. Early developmental intervention programmes provided post hospital discharge to prevent motor and cognitive impairment in preterm infants. Cochrane Database Syst Rev. (2015) 11:CD005495. 10.1002/14651858.CD005495.pub4 26597166 PMC8612699

[21] Groh AM Fearon RMP van IJzendoorn MH Bakermans-Kranenburg MJ Roisman GI. Attachment in the early life course: meta-analytic evidence for its role in socioemotional development. Child Dev Perspect. (2017) 11:70–6. 10.1111/cdep.12213

[22] Festante F Antonelli C Chorna O Corsi G Guzzetta A. Parent-infant interactions during the first year of life in infants at high risk for cerebral palsy: a systematic review of the literature. Neural Plast. (2019) 2019:5759694. 10.1155/2019/5759694 31178902 PMC6501141

[23] Slonims V McConachie H. Analysis of mother-infant interaction in infants with Down syndrome and typically developing infants. Am J Mental Retard. (2006) 111:273–89. 10.1352/0895-8017( 2006) 111273:AOMIII2.0.CO 16792429

[24] Wan MW Green J Scott J. A systematic review of parent-infant interaction in infants at risk of autism. Autism. (2019) 23:811–20. 10.1177/1362361318777484 29992838

[25] Adamson LB Deckner DF Bakeman R. Early interests and joint engagement in typical development, autism, and Down syndrome. J Autism Dev Disord. (2010) 40:665–76. 10.1007/s10803-009-0914-1 20012678 PMC2873143

[26] Field T. Expressivity in physically and emotionally handicapped children. In: Lewis M, Sullivan MW, editors. Emotional Development in Atypical Children. New York, NY: Psychology Press (1996). p. 1–28.

[27] Barfoot J Meredith P Ziviani J Whittingham K. Parent-child interactions and children with cerebral palsy: an exploratory study investigating emotional availability, functional ability, and parent distress. Child Care Health Dev. (2017) 43:812–22. 10.1111/cch.12493 28737004

[28] Bernard K Nissim G Vaccaro S Harris JL Lindhiem O. Association between maternal depression and maternal sensitivity from birth to 12 months: a meta-analysis. Attach Hum Dev. (2018) 20:578–99. 10.1080/14616734.2018.1430839 29374991

[29] Pennington L McConachie H. Predicting patterns of interaction between children with cerebral palsy and their mothers. Dev Med Child Neurol. (2001) 43:83–90. 10.1017/S0012162201000147 11221909

[30] Feldman R. From biological rhythms to social rhythms: physiological precursors of mother-infant synchrony. Dev Psychol. (2006) 42:175–88. 10.1037/0012-1649.42.1.175 16420127

[31] Harel-Gadassi A Friedlander E Yaari M Bar-Oz B Eventov-Friedman S Mankuta D . Do developmental and temperamental characteristics mediate the association between preterm birth and the quality of mother-child interaction? Infant Behav Dev. (2020) 58:101421. 10.1016/j.infbeh.2020.101421 32135402

[32] Vreeswijk CMJM Maas AJBM van Bakel HJA. Parental representations: a systematic review of the working model of the child interview. Infant Ment Health J. (2012) 33:314–28. 10.1002/imhj.20337 28520281

[33] Feldman R. Maternal versus child risk and the development of parent-child and family relationships in five high-risk populations. Dev Psychopathol. (2007) 19:293–312. 10.1017/S0954579407070150 17459172

[34] Cabrera NJ Volling BL Barr R. Fathers are parents, too! Widening the lens on parenting for children's development. Child Dev Perspect. (2018) 12:152–7. 10.1111/cdep.12275

[35] Willerslev-Olsen M Lorentzen J Røhder K Ritterband-Rosenbaum A Justiniano M Guzzetta A . CONTRACT (copenhagen neuroplastic training against contractures in toddlers): protocol of an open-label randomized clinical trial with blinded assessment for prevention of contractures in infants with high risk of cerebral palsy. BMJ Open. (2021) 11:e044674. 10.1136/bmjopen-2020-044674 34230015 PMC8261878

[36] Feldman R. Coding Interactive Behavior Manual. Ramat-Gan: Bar Ilan University (1998).

[37] Feldman R Bamberger E Kanat-Maymon Y. Parent-specific reciprocity from infancy to adolescence shapes children's social competence and dialogical skills. Attach Hum Dev. (2013) 15:407–23. 10.1080/14616734.2013.782650 23544455

[38] Branger MCE Emmen RAG Woudstra MJ Alink LRA Mesman J. Context matters: maternal and paternal sensitivity to infants in four settings. J Fam Psychol. (2019) 33:851–6. 10.1037/fam0000562 31318265

[39] Maas AJ Vreeswijk CM van Bakel HJ. Effect of situation on mother-infant interaction. Infant Behav Dev. (2013) 36:42–9. 10.1016/j.infbeh.2012.10.006 23261788

[40] Squires J Bricker D Twombly E. Ages and Stages Questionnaires^®^: Social-Emotional, Second Edition (ASQ®:SE-2). Baltimore, MD: Brookes Publishing (2015).

[41] Pontoppidan M Niss NK Pejtersen JH Julian MM Vaever MS. Parent report measures of infant and toddler social-emotional development: a systematic review. Fam Pract. (2017) 34:127–37. 10.1093/fampra/cmx003 28158522

[42] Bayley N. Bayley Scales of Infant Development, 3rd ed. San Antonio, TX: Harcourt Assessment Inc (2006).

[43] Krogh MT Væver MS. A longitudinal study of the predictive validity of the Bayley-III scales and subtests. Eur J Dev Psychol. (2019) 16:727–38. 10.1080/17405629.2018.1485563

[44] Krogh MT Væver MS Harder S Køppe S. Cultural differences in infant development during the first year: a study of Danish infants assessed by the Bayley-III and compared to the American norms. Eur J Dev Psychol. (2021) 9:730–6. 10.1080/17405629.2012.688101

[45] Zeanah CH Benoit D Hirschberg L Barton ML. Working Model of the Child Interview Providence, RI: Brown University Program in Medicine, (1993).

[46] Crawford A Benoit D. Caregivers' disrupted representations of the unborn child predict later infant-caregiver disorganized attachment and disrupted interactions. Infant Ment Health J. (2009) 30:124–44. 10.1002/imhj.20207 28636176

[47] George C Solomon J. The caregiving system: a behavioral systems approach to parenting. In: Cassidy J, Shaver PR, editors. Handbook of Attachment: Theory, Research, and Clinical Applications. New York, NY: The Guilford Press (2008). p. 833–56.

[48] Korja R Ahlqvist-Bjorkroth S Savonlahti E Stolt S Haataja L Lapinleimu H . Relations between maternal attachment representations and the quality of mother-infant interaction in preterm and full-term infants. Infant Behav Dev. (2010) 33:330–6. 10.1016/j.infbeh.2010.03.010 20417970

[49] Schechter DS Coates SW Kaminer T Coots T Zeanah CH Jr. Davies M . Distorted maternal mental representations and atypical behavior in a clinical sample of violence-exposed mothers and their toddlers. J Trauma Dissociat. (2008) 9:123–47. 10.1080/15299730802045666 18985165 PMC2577290

[50] Sokolowski MS Hans SL Bernstein VJ Cox SM. Mothers' representations of their infants and parenting behavior: associations with personal and social-contextual variables in a high-risk sample. Infant Ment Health J. (2007) 28:344–65. 10.1002/imhj.20140 28640464

[51] Benoit D Parker KC Zeanah CH. Mothers' representations of their infants assessed prenatally: stability and association with infants' attachment classifications. J Child Psychol Psychiatry Allied Discip. (1997) 38:307–13. 10.1111/j.1469-7610.1997.tb01515.x 9232477

[52] Zeanah CH Benoit D Hirshberg L Barton M Regan C. Mothers' representations of their infants are concordant with infant attachment classifications. Dev Issues Psychiatry Psychol. (1994) 1:1–14.

[53] Antony MM Bieling PJ Cox BJ Enns MW Swinson RP. Psychometric properties of the 42-item and 21-item versions of the depression anxiety stress scales in clinical groups and a community sample. Psychol Assess. (1998) 10:176–81. 10.1037/1040-3590.10.2.176

[54] Lovibond PF Lovibond SH. The structure of negative emotional states: comparison of the depression anxiety stress scales (DASS) with the beck depression and anxiety inventories. Behav Res Ther. (1995) 33:335–43. 10.1016/0005-7967(94)00075-U 7726811

[55] Morgan C Novak I Dale RC Guzzetta A Badawi N. Single blind randomised controlled trial of GAME (goals - activity - motor enrichment) in infants at high risk of cerebral palsy. Res Dev Disabil. (2016) 55:256–67. 10.1016/j.ridd.2016.04.005 27164480

[56] Henry JD Crawford JR. The short-form version of the depression anxiety stress scales (DASS-21): construct validity and normative data in a large non-clinical sample. Br J Clin Psychol. (2005) 44:227–39. 10.1348/014466505X29657 16004657

[57] Osman A Wong JL Bagge CL Freedenthal S Gutierrez PM Lozano G. The depression anxiety stress scales-21 (DASS-21): further examination of dimensions, scale reliability, and correlates. J Clin Psychol. (2012) 68:1322–38. 10.1002/jclp.21908 22930477

[58] Sinclair SJ Siefert CJ Slavin-Mulford JM Stein MB Renna M Blais MA. Psychometric evaluation and normative data for the depression, anxiety, and stress scales-21 (DASS-21) in a nonclinical sample of U.S. adults. Eval Health Profess. (2012) 35:259–79. 10.1177/0163278711424282 22008979

[59] Ayers S Wright DB Thornton A. Development of a measure of postpartum PTSD: the City Birth Trauma scale. Front Psychiatry. (2018) 9:409. 10.3389/fpsyt.2018.00409 30279664 PMC6153962

[60] Caparros-Gonzalez RA Romero-Gonzalez B Peralta-Ramirez MI Ayers S Galan-Paredes A Caracuel-Romero A. Assessment of posttraumatic stress disorder among women after childbirth using the City Birth Trauma Scale in Spain. Psychol Trauma Theory Res Pract Policy. (2021) 13:545–54. 10.1037/tra0001007 33507792

[61] Nakic Rados S Matijas M Kuhar L Andelinovic M Ayers S. Measuring and conceptualizing PTSD following childbirth: validation of the City Birth Trauma Scale. Psychol Trauma Theory Res Pract Policy. (2020) 12:147–55. 10.1037/tra0000501 31368743

[62] Abadin RR. Parenting Stress Index. 4th ed. Lutz, FL: PAR (2012).

[63] Barroso NE Hungerford GM Garcia D Graziano PA Bagner DM. Psychometric properties of the parenting stress index-short form (PSI-SF) in a high-risk sample of mothers and their infants. Psychol Assess. (2016) 28:1331–5. 10.1037/pas0000257 26595220 PMC4877285

[64] Ketelaar M Volman MJ Gorter JW Vermeer A. Stress in parents of children with cerebral palsy: what sources of stress are we talking about? Child Care Health Dev. (2008) 34:825–9. 10.1111/j.1365-2214.2008.00876.x 18959579

[65] Cox JL Holden JM Sagovsky R. Detection of postnatal depression. Development of the 10-item Edinburgh postnatal depression scale. Br J Psychiatry. (1987) 150:782–6. 10.1192/bjp.150.6.782 3651732

[66] Smith-Nielsen J Matthey S Lange T Vaever MS. Validation of the Edinburgh postnatal depression scale against both DSM-5 and ICD-10 diagnostic criteria for depression. BMC Psychiatry. (2018) 18:393. 10.1186/s12888-018-1965-7 30572867 PMC6302501

[67] Landgraf J Abetz L. The Infant/Toddler Child Health Questionnaire: Conceptual Framework, Logic Content, and Preliminary Psychometric Results. Boston: Health Act (1994).

[68] World Health Organization. Constitution. (2021). Available online at: https://www.who.int/about/governance/constitution

[69] Raat H Landgraf JM Oostenbrink R Moll HA Essink-Bot ML. Reliability and validity of the infant and toddler quality of life questionnaire (ITQOL) in a general population and respiratory disease sample. Qual Life Res. (2007) 16:445–60. 10.1007/s11136-006-9134-8 17111231 PMC2792359

[70] Darrah J Piper M Watt MJ. Assessment of gross motor skills of at-risk infants: predictive validity of the alberta infant motor scale. Dev Med Child Neurol. (1998) 40:485–91. 10.1111/j.1469-8749.1998.tb15399.x 9698062

[71] Sameroff A. The Transactional Model of Development: How Children and Contexts Shape Each Other. Washington, DC: American Psychological Association (2009).

[72] Sroufe LA Egeland B Carlson EA Collins WA. The Development of the Person: the Minnesota Study of Risk and Adaptation from Birth to Adulthood. New York, NY: The Guilford Press (2009).

[73] Spiker D Boyce GC Boyce LK. Parent-child interactions when young children have disabilities. Int Rev Res Ment Retard. (2002) 25:35–70. 10.1016/S0074-7750(02)80005-226647404

[74] Poehlmann J Fiese BH. Parent-infant interaction as a mediator of the relation between neonatal risk status and 12-month cognitive development. Infant Behav Dev. (2001) 24:171–88. 10.1016/S0163-6383(01)00073-X

[75] Liu J Bann C Lester B Tronick E Das A Lagasse L . Neonatal neurobehavior predicts medical and behavioral outcome. Pediatrics. (2010) 125:e90–98. 10.1542/peds.2009-0204 19969621 PMC2873896

[76] Barlow JH Cullen-Powell LA Cheshire A. Psychological well-being among mothers of children with cerebral palsy. Early Child Dev Care. (2006) 176:421–8. 10.1080/0300443042000313403

[77] Barreto TM Bento MN Barreto TM Jagersbacher JG Jones NS Lucena R . Prevalence of depression, anxiety, and substance-related disorders in parents of children with cerebral palsy: a systematic review. Dev Med Child Neurol. (2020) 62:163–8. 10.1111/dmcn.14321 31381150

[78] Cheshire A Barlow JH Powell LA. The psychosocial well-being of parents of children with cerebral palsy: a comparison study. Disabil Rehabil. (2010) 32:1673–7. 10.3109/09638281003649920 20178413

[79] Pousada M Guillamón N Hernández-Encuentra E Muñoz E Redolar D Boixadós M . Impact of caring for a child with cerebral palsy on the quality of life of parents: a systematic review of the literature. J Dev Phys Disabil. (2013) 25:545–77. 10.1007/s10882-013-9332-6

[80] Treyvaud K Anderson VA Lee KJ Woodward LJ Newnham C Inder TE . Parental mental health and early social-emotional development of children born very preterm. J Pediatr Psychol. (2010) 35:768–77. 10.1093/jpepsy/jsp109 19955253

[81] Korja R Savonlahti E Haataja L Lapinleimu H Manninen H Piha J . Attachment representations in mothers of preterm infants. Infant Behav Dev. (2009) 32:305–11. 10.1016/j.infbeh.2009.04.003 19446341

[82] Tooten A Hall RA Hoffenkamp HN Braeken J Vingerhoets AJ van Bakel HJ. Maternal and paternal infant representations: a comparison between parents of term and preterm infants. Infant Behav Dev. (2014) 37:366–79. 10.1016/j.infbeh.2014.05.004 24887535

[83] Hall RA Hoffenkamp HN Tooten A Braeken J Vingerhoets AJ van Bakel HJ. Longitudinal associations between maternal disrupted representations, maternal interactive behavior and infant attachment: a comparison between full-term and preterm dyads. Child Psychiatry Hum Dev. (2015) 46:320–31. 10.1007/s10578-014-0473-3 24875043

[84] Borghini A Pierrehumbert B Miljkovitch R Muller-Nix C Forcada-Guex M Ansermet F. Mother's attachment representations of their premature infant at 6 and 18 months after birth. Infant Ment Health J. (2006) 27:494–508. 10.1002/imhj.20103 28640398

[85] Hoffenkamp HN Braeken J Hall RA Tooten A Vingerhoets AJ van Bakel HJ. Parenting in complex conditions: does preterm birth provide a context for the development of less optimal parental behavior? J Pediatr Psychol. (2015) 40:559–71. 10.1093/jpepsy/jsv007 25699688

[86] Meijssen D Wolf MJ van Bakel H Koldewijn K Kok J van Baar A. Maternal attachment representations after very preterm birth and the effect of early intervention. Infant Behav Dev. (2011) 34:72–80. 10.1016/j.infbeh.2010.09.009 21067812

